# Khorasan Disease: Prevalence of HTLV-I Associated Myelopathy/Tropical Spastic Paraparesis (HAM/TSP) In West Azarbaijan from 2004 to 2007

**Published:** 2011-06-01

**Authors:** R Boostani, A Mellat Ardakani, H Ashrafi

**Affiliations:** 1 Department of Neurology, Mashhad University of Medical Sciences, Mashhad, Iran; 2 Department of Neurology, Urmia University of Medical Sciences, Urmia, Iran

**Keywords:** HTLV-I, HAM/TSP, Spastic paraparesis, Iran

## Abstract

**Background:**

Because of the low prevalence of Human T Lymphotropic Virus type I (HTLV-I) in comparison with Khorasan Province, considering HTLV-I as an etiology of spastic paraparesia, it may be neglected in evaluation of spastic paraparesis in the other regions of Iran. Some reports of spastic paraparetic patients due to HTLV-I infection in West Azarbaijan, caused us to reconsider the importance of HTLV-I epidemiology in the other areas of the country.

**Methods:**

All spastic paraparetic patients who referred to Motahari and Imam Khomeini educational hospitals of Urmia from September 2004 to September 2007 were assessed for HTLV-I infection antibodies.

**Results:**

In our 3 years study, 11 cases were diagnosed as Human T Lymphotropic Virus type I Associated Myelopathy/Tropical Spastic Paraparesis (HAM/TSP, 2 males and 9 females).The mean age of patients at the time of diagnosis was 45.8 years. Dorsal and cervical MRI of all patients was normal. Serum Enzyme-Linked Immuno-Sorbent Assay (ELISA) and Western blot (WB) for anti HTLV-I antibody in all patients was positive. Four patients underwent for lumbar puncture in which were normal in respect of cells and biochemistry, but positive for anti-HLTLV-I antibodies.

**Conclusion:**

HAM/TSP detection in West Azarbaijan in spite of its long distance from Khorasan Province shows the importance of anti-HTLV-I Ab assay in the blood and CSF of every spastic paraparetic patient all over the country.

## Introduction

During the past 25 years, Human T Lymphotropic Virus type I (HTLV-I) epidemiology has been studied many times getting more advanced day by day. Geographical distribution of virus in the world was previously demonstrated. Although several studies showed the high prevalence of HTLV-I in Japan southwest, however the prevalence of virus in adjacent countries like Korea, China, Eastern Russia and Khorasan in Iran is low.[1] HTLV-I can induce several diseases such as Adult T Cell Leukemia (ATL), Human T Lymphotropic Virus type I Associated Myelopathy/Tropical Spastic Paraparesis (HAM/TSP), sensori-motor polyneuropathy and optic neuritis.[2][3][4] Many cases of disease due to transmission are well established and the disease is known as a sexual transmitted disease.[5] The majority of epidemiologic studies are based on serologic screening for anti HTLV-I anti body (Ab) by using blood Enzyme-Linked Immuno-Sorbent Assay (ELISA) and is confirmed by Western Blot (WB) test.[6] Polymerase Chain Reaction (PCR) is used to confirm HTLV-I typing diagnosis.[7] The presence of endemic area in north-east of Iran (especially Mashhad, Neishaboor and adjacent areas), doubled the importance of HTLV-I epidemiologic studies in Iran. So in this area of the country every spastic paraparetic patient who referred is evaluated for anti HTLV-I Ab,but many reports indicate the existence of HAM/TSP in the other regions of the country such as West Azarbaijan and it challenged us to evaluate cumulative prevalence of this disease in the West Azarbaijan and discover the importance of HTLV-I epidemiology around Iran.

## Materials and Methods

All patients presented with spastic paraparesia and referred to Motrahari and Imam Khomeini hospitals in Urmia, from September 2004 to September 2007 were assessed for anti HTLV-I Ab.

All patients after systemic and neurologic examinations underwent dorsal and cervical Magnetic Resonance Imaging (MRI). After ruling out of compressive lesions all patients’ blood were tested for anti HTLV-I Ab with ELISA and WB methods. Only four patients allowed us to do lumbar puncture for Cerebro- spinal Fluid (CSF) analysis (cell, biochemistry and anti HTLV-I Ab). The results are presented in Table 1 and Table 2.

**Table 1:** Presenting symptoms of patients diagnosed as HAM-TSP

**Gait disturbance**	**Urinary disturbance (frequency and incontinence)**	**Low back and lower limb pain**	**Paresthesia**
11/11	10/11	7/11	6/11

**Table 2:** Objective findings in patients diagnosed as HAM-TSP

**Spastic gait**	**Babinski sign**	**Weakness**	**Hyper-reflexia**		**Sensory impairment (lower limb)**	
			**Upper limb**	**Lower limb**	**Pain and temperature**	**Position and properioceptive**
11/11	11/11	11/11	6/11	11/11	2/11	7/11

## Results

Eleven patients (2 males and 9 women) were diagnosed as HAM/TSP. The mean age for diagnosis was 45.8 years and for beginning of symptoms based on history was 36.3 years old. Three patients had a history of blood transfusion (all were women). Three patients were under treatment for primary progressive multiple sclerosis. All patients underwent dorsal and cervical MRI which were normal.

Using ELISA and WB methods showed anti-HTLVI Ab in all patients. Only four patients underwent lumbar puncture in which all were normal for cell and biochemistry but positive for anti-HTLV-I antibodies.

## Discussion

Epidemiologic studies have been done over the past 25 year's demonestrating worldwide distribution of HTLV-I with different prevalence rates. Because of wide distribution of HTLV-I infection all over the world, doing epidemiological studies in every region (even non-endemic areas) is essential. In some nonendemic areas such as rural West African[8] and South American Indians (Mapuches) from Chile,[9] the results were interesting. These studies have been done among high risk groups and at risk populations such as pregnant women.[10] In Iran, these studies showed some endemic areas in Khorasan Province. Our findings showed that there was a least approximately 10 years interval between the appearance of the symptoms and the diagnosis of disease, indicating that there has been no sufficient notice in finding the virus in the other regions of the country such as Azarbaijan Province. Finding three cases of HTLV-I positive people, due to blood transfusion indicates the importance of screening for the virus before any blood transfusion. This fact is not supported the need for HTLV-I/II antenatal screening in some non-endemic areas such as Greece.[11]

A positive blood result for HTLV-I by ELISA in all patients indicates the high sensitivity of this test and repeatedly, proving the fact that ELISA is the best screening test in HTLV-I assay. Normal dorsal and cervical MRI in patients who referred to us with spastic paraparesia, reinforces the suspicion for viral and infectious etiologies such as HTLV-I. The presence of upper motor neuron signs with less severity in upper limbs is an interesting finding in which should be considered in HAM/TSP.

Reporting 11 HAM/TSP patients in West Azarbaijan during a three years interval indicates the distribution of HTLV-I in Iran that remind us of the necessity for evaluation of any patient who refer with spastic paraparesia or spastic tetraparesia for HTLV-I.

Regarding the possibility of HTLV-I transmission by blood transfusion, assessing the patients with the history of blood transfusion in past decades should be considered. Finally we are emphasizing that HAM/TSP should be considered in patients with subjective complains such as urinary incontinence or frequency, distal paresthesia, dysesthesia, muscle stiffness and gait disturbances.
